# The Molecular Mechanism Regulating Flavonoid Production in *Rhododendron chrysanthum* Pall. Against UV-B Damage Is Mediated by *RcTRP5*

**DOI:** 10.3390/ijms252413383

**Published:** 2024-12-13

**Authors:** Fushuai Gong, Jinhao Meng, Hongwei Xu, Xiaofu Zhou

**Affiliations:** Jilin Provincial Key Laboratory of Plant Resource Science and Green Production, Jilin Normal University, Siping 136000, China; 13578825948@163.com (F.G.);

**Keywords:** *RcTRP5*, flavonoid biosynthesis, *R. chrysanthum*, UV-B stress

## Abstract

Elevated levels of reactive oxygen species (ROS) are caused by ultraviolet B radiation (UV-B) stress. In response, plants strengthen their cell membranes, impeding photosynthesis. Additionally, UV-B stress initiates oxidative stress within the antioxidant defense system and alters secondary metabolism, particularly by increasing the quantity of UV-absorbing compounds such as flavonoids. The v-myb avian myeloblastosis viral oncogene homolog (MYB) transcription factor (TF) may participate in a plant’s response to UV-B damage through its regulation of flavonoid biosynthesis. In this study, we discovered that the photosynthetic activity of *Rhododendron chrysanthum* Pall. (*R. chrysanthum*) decreased when assessing parameters of chlorophyll (PSII) fluorescence parameters under UV-B stress. Concurrently, antioxidant system enzyme expression increased under UV-B exposure. A multi-omics data analysis revealed that acetylation at the K68 site of the RcTRP5 (telomeric repeat binding protein of *Rhododendron chrysanthum* Pall.) transcription factor was upregulated. This acetylation modification of RcTRP5 activates the antioxidant enzyme system, leading to elevated expression levels of peroxidase (POD), superoxide dismutase (SOD), and catalase (CAT). Upregulation is also observed at the K95 site of the chalcone isomerase (CHI) enzyme and the K178 site of the anthocyanidin synthase (ANS) enzyme. We hypothesize that *RcTRP5* influences acetylation modifications of CHI and ANS in flavonoid biosynthesis, thereby indirectly regulating flavonoid production. This study demonstrates that *R. chrysanthum* can be protected from UV-B stress by accumulating flavonoids. This could serve as a useful strategy for enhancing the plant’s flavonoid content and provide a valuable reference for research on the metabolic regulation mechanisms of other secondary substances.

## 1. Introduction

*R. chrysanthum* is a perennial evergreen dwarf shrub belonging to the Ericaceae family, with a height ranging from 10 to 50 cm. It is an endangered species. In China, it is primarily distributed in the alpine tundra zone of the Changbai Mountains, at altitudes ranging from 1300 to 2506 m [1]. Due to the strong ultraviolet radiation, low temperatures, and other harsh environmental factors in the Changbai Mountains, *R. chrysanthum* has evolved the ability to resist such radiation, temperatures, and other abiotic stresses. Therefore, *R. chrysanthum* represents a special botanical resource for studying plant resilience [2]. Meanwhile, our previous studies have provided sufficient physiological evidence for this species’ strong resistance to UV-B stress [3,4].

Widespread hydrochlorofluorocarbon (HCFC) pollution has recently entered the stratosphere, speeding up the thinning of the ozone layer and accelerating the rate at which ozone molecules are destroyed. This, in turn, has led to an increase in UV-B radiation worldwide within the solar ultraviolet spectrum, exerting far-reaching effects on all life on Earth [5]. UV-B stress impacts plant physiological and biochemical systems, which is evident from increased ROS levels and decreased photosynthetic efficiency. Chlorophyll fluorescence parameters serve as indicators of photosystem functioning and reflect a plant’s actual photosynthetic efficiency [6]. The chlorophyll fluorescence kinetic curve describes the quenching process when the PSII reaction center is fully closed, resulting in a progressive decrease in fluorescence intensity. This process involves the sequential electron transfer on the electron transport chain to NADP^+^ for NADPH generation. It reflects the proportion of light energy absorbed by the photosynthetic system in light environments and its potential photochemical activity. Additionally, plants enhance the production of enzymatic antioxidants, such as ascorbate peroxidase (APX), SOD, CAT, and POD, to mitigate ROS damage.

In the field of plant science, the phenomenon of UV-B stress-induced accumulation of UV-absorbing compounds has been extensively examined. These compounds are primarily flavonoids but also include other phenolic complexes, such as those of flavonoids with erucic acid esters. These chemicals are produced by plants in an effort to shield vulnerable tissues from UV-B irradiation. Studies on how UV-absorbing substances respond to UV-B stress have gained increasing attention in recent years. These studies, on kale (*Brassica oleracea*) [7] and rice (*Oryza sativa*) [8], have demonstrated that plants can adapt to UV-B stress by modifying the flavonoid biosynthetic pathway.

Many plants possess a well-characterized mechanism for flavonoid biosynthesis that is triggered by UV-B radiation [9]. In response to UV-B light, the UV-B photoreceptor UV RESISTANT LOCUS 8 (UVR8) in *Arabidopsis thaliana* interacts with CONSTITUTIVE PHOTOMORPHOGENIC 1 (COP1) to activate UV-B signaling and initiate the UV-B acclimation process [10]. Subsequently, COP1 activates ELONGATED HYPOCOTYL 5 (HY5), a basic leucine zipper (bZIP) transcription factor, which in turn promotes anthocyanin accumulation by regulating the expression of MYB transcription factors and genes involved in anthocyanin biosynthesis [11].

The MYB protein superfamily encompasses transcription factors that respond to diverse stressors and regulate the synthesis of secondary metabolites [12]. Notably, activators of anthocyanin biosynthesis include VbMYBA in *Vaccinium bracteatum* [13], *VmMYBA1* and *VmMYBA2* in *Vaccinium myrtillus* [14], and *VcMYBA* in *blueberries* [15]. Conversely, *FaMYB1* in strawberries (*Fragaria* × *ananassa*) inhibits anthocyanin accumulation [16]. In *Arabidopsis thaliana* exposed to UV-B light, *AtMYB4* is downregulated and *AtMYB75* is upregulated [17,18]. However, further research is required to elucidate the response of MYB pathway genes to UV-B radiation.

In the process of acetylation modification, acetyl groups are appended to lysine residues of proteins, marking it as a ubiquitous post-translational modification. This modification regulates a range of biochemical processes, including transcriptional activity, enzyme activity, and chromatin structure [19]. By influencing the stability, affinity, and protein–protein interactions of key enzymes involved in secondary metabolic pathways, acetylation alterations can modulate the degree of protein acetylation, thereby affecting the production of secondary metabolites. Notably, the biosynthesis of anthocyanins in plants, such as *Arabidopsis thaliana*, necessitates acetylation changes [20].

## 2. Results

### 2.1. Analysis of R. chrysanthum Fluorescence Imaging Parameters Under UV-B Stress

**Figure 1:**
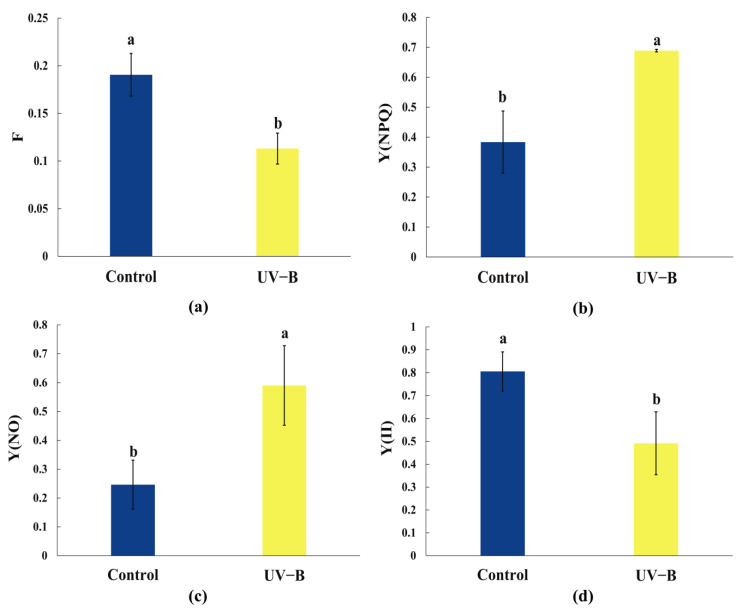
Trends in *R. chrysanthum’s* photosynthetic characteristics under UV-B stress: (**a**–**d**) real-time fluorescence actual, quantum yield of modulatable quenching in PSII, quantum yield of non-modulatable quenching in PSII, and photosynthetic efficiency of PSII, respectively. The data represent the mean ± SD for *n* = 3. A significant difference among treatments at *p* < 0.05 is indicated by different letters (a, b).

Figure 1 presents the numerical variations of chlorophyll fluorescence parameters F (real-time fluorescence actual), Y(NPQ) (quantum yield of modulatable quenching in PSII), Y(NO) (quantum yield of non-modulatable quenching in PSII), and Y(II) (photosynthetic efficiency of PSII) in *R. chrysanthum* under UV-B stress. Firstly, it was observed that both F and Y(II) exhibited significant declines under UV-B stress, indicating adverse effects on photosynthesis. It is important to note that under normal growth conditions, the photochemical reaction, heat dissipation, and chlorophyll fluorescence each have a fixed quantum yield of 1, reflecting their competitive nature. Normally, a small portion of absorbed light energy is utilized for heat dissipation and fluorescence, while the majority is channeled through photochemical processes. However, under environmental stress such as UV-B exposure, photosynthetic efficiency decreases.

The plant reaction center employs non-photochemical quenching (NPQ) to dissipate excess light energy, thereby mitigating photoinhibition and photodamage to PSII, leading to a reduction in the chlorophyll fluorescence parameter F of *R. chrysanthum* (Figure 1a). Under UV-B stress, *R. chrysanthum* exhibited photoprotection, reflected by an increase in Y(NPQ), which indicates enhanced heat dissipation as a protective mechanism (Figure 1b). Simultaneously, Y(NO), a metric for light damage, increased, suggesting a limitation in photosynthetic activity due to UV-B stress (Figure 1c). Furthermore, the true photochemical quantum yield, represented by Y(II), significantly decreased under UV-B stress, clearly demonstrating the stressor’s inhibitory effect on photosynthesis (Figure 1d).

### 2.2. UV-B Stress Treatment and Its Effects on the Expression of Flavonoid Compounds

**Figure 2:**
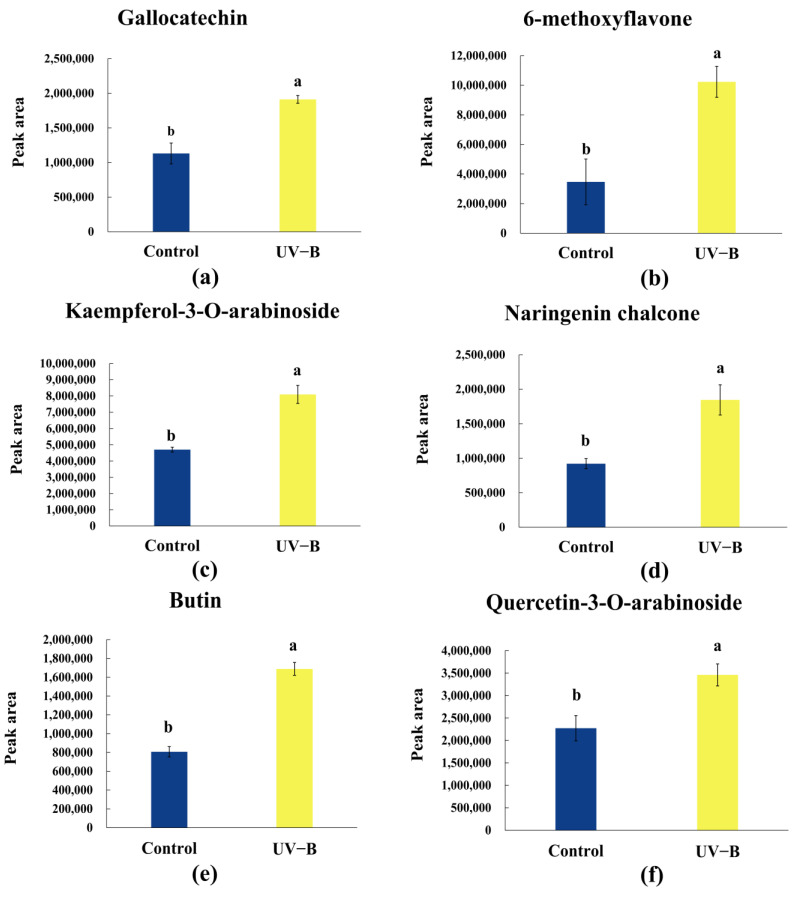
Flavonoid trends in six *R. chrysanthum* species in response to UV-B exposure: (**a**–**f**) gallocatechin, 6-methoxyflavone, kaempferol-3-O-arabinoside, naringenin chalcone, butin, and quercetin-3-O-arabinoside, respectively. The data represent the mean ± SD for *n* = 3. A significant difference among treatments at *p* < 0.05 is indicated by different letters (a, b).

UV-B stress not only inhibits photosynthesis but also reduces the levels of secondary metabolites in *R. chrysanthum*. A metabolomics analysis of samples exposed to normal light versus UV-B stress identified a total of 2148 metabolites, including 487 flavonoids, with significant changes observed in 164 flavonoids. These flavonoids are pivotal for elucidating the molecular mechanisms of *R. chrysanthum*’s response to UV-B stress. Notably, all six flavonoids with the most significant changes exhibited an upward trend in expression (Figure 2).

Our findings suggest that *R. chrysanthum* responds to UV-B stress by increasing flavonoid expression, potentially as a mechanism to counteract UV-B radiation damage by producing UV-absorbing compounds.

### 2.3. RcTRP5′s Reaction to UV-B Stress

For transcriptomics analysis, we generated 76.65 GB of data using the DNBSEQ platform and annotated transcription factors based on the NR database and the Arabidopsis information.

**Figure 3:**
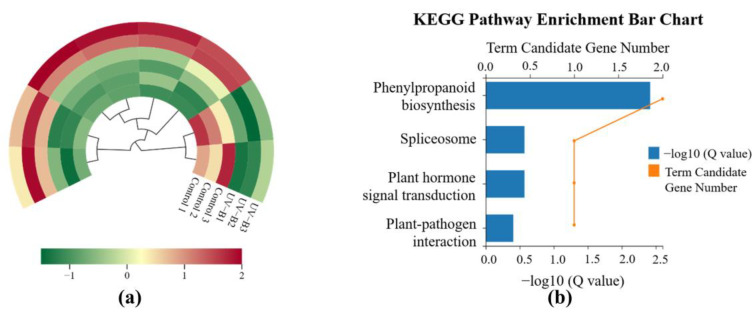
Enrichment analysis of MYB transcription factors significantly altered by UV-B stress in the *R. chrysanthum*: (**a**) there were notable variations in the expression levels of eight MYB transcription factors in rhododendron that respond to UV-B stress; red indicates higher expression levels and green lower expression levels; (**b**) eight MYB transcription factors in the *R. chrysanthum* were analyzed for enrichment.

Transcriptome experiments revealed 28 types of transcription factors in *R. chrysanthum* under UV-B exposure. Notably, the MYB transcription factor family was the most abundant and played a pivotal role in response to UV-B stress. Further differential screening identified eight significantly altered transcription factors: six were upregulated and the other two were downregulated (Figure 3a). Enrichment analysis of these eight transcription factors indicated that only one, *RcTRP5*, was involved, with the phenylpropanoid biosynthesis pathway being the only significantly enriched pathway (Figure 3b). Detailed information on *RcTRP5* is provided in Table S1.

### 2.4. Changes in the Antioxidant Defense System of the R. chrysanthum Under UV-B Stress

**Figure 4:**
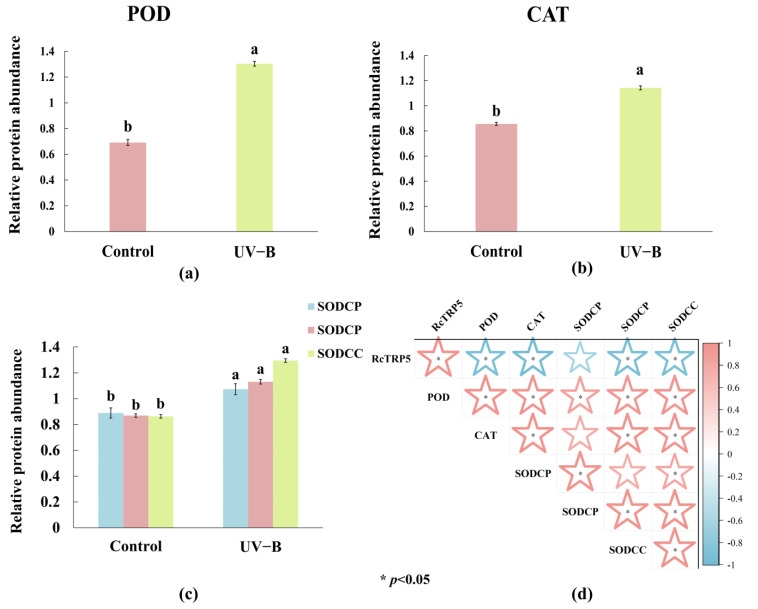
Response of antioxidant enzyme system of *R. chrysanthum* to UV-B stress and correlation analysis with *RcTRP5*: (**a**–**c**) POD: peroxidase; CAT1: catalase isozyme 1; SODCC: superoxide dismutase; SODCP: superoxide dismutase; (**d**) the more pinkish the color, the stronger the positive correlation; the more bluish the color, the stronger the negative correlation. The data represent the mean ± SD for *n* = 3. A significant difference among treatments at *p* < 0.05 is indicated by different letters (a, b). Asterisks denote treatments with significant changes (*p* < 0.05).

*R. chrysanthum* exhibits a synergistic response to UV-B exposure, involving antioxidant enzyme systems and MYB transcription factors. Proteomics assays identified 6378 proteins, with 5191 being quantifiable and 505 being significantly altered under UV-B stress. Notably, POD, CAT, and SOD showed significant changes (Figure 4a–c). During UV-B stress, POD increased, facilitating the breakdown of hydrogen peroxide (H_2_O_2_) and other peroxides into less toxic compounds while also aiding in redox balance restoration by catalyzing ROS decomposition. CAT specifically catalyzes the conversion of H_2_O_2_ into oxygen and water, and SOD catalyzes the disproportionation of O^2−^ superoxide anion radicals, generating oxygen and H_2_O_2_. These enzymes synergistically resist oxidative stress under UV-B. Correlation analysis with *RcTRP5*, identified through transcriptomics, revealed a substantial negative association between *RcTRP5* and the antioxidant enzyme system, suggesting that *RcTRP5* may negatively regulate the antioxidant enzyme system in response to UV-B damage.

### 2.5. Variations in the R. chrysanthum’s Flavonoid Biosynthesis Under UV-B Stress

This study focuses on flavonoid biosynthesis under UV-B stress, with reference to previous findings on lignin biosynthesis’s response to UV-B [21]. The reaction of *R. chrysanthum* to UV-B involves the cooperative actions of flavonoid biosynthesis pathways and MYB transcription factors. To investigate the increased expression of genes and metabolites in the flavonoid biosynthesis pathway, comprehensive targeted metabolomics assays were conducted. Using the UPLC-MS/MS platform and a self-constructed database from Jingjie PTM BioLab (Hangzhou, China) Co., Ltd., we detected 2148 metabolites, of which 706 were significantly altered under UV-B stress.

**Figure 5:**
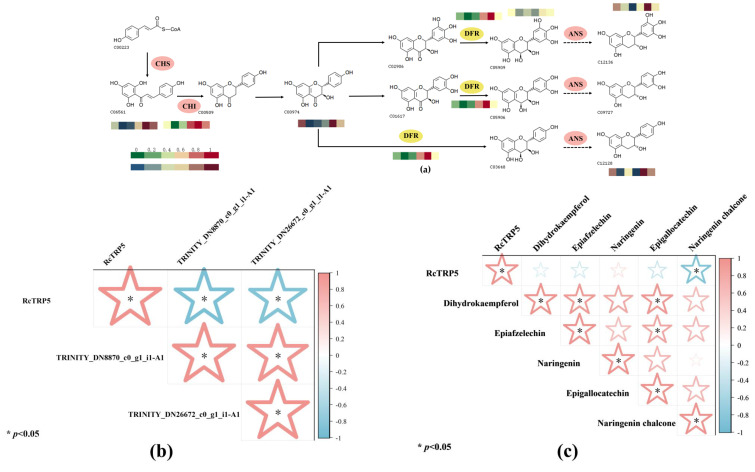
*R. chrysanthum* regulates the production of flavonoids: (**a**) data on metabolite content and enzyme gene expression were shown on a heat map after being normalized using the formula (Xi − min(x))/(max(x) − min(x)). Heatmaps with dark-red and dark-blue hues show changes in metabolite expression, with redder hues denoting higher expression and bluer hues denoting lower expression. Red and green heatmaps show changes in the expression of enzyme genes; redder hues denote higher expression, while greener hues denote lower expression; (**b**,**c**) the more pinkish the color, the stronger the positive correlation; the more bluish the color, the stronger the negative correlation. For *n* = 3, the data are the mean ± SD. Asterisks denote treatments with significant changes (*p* < 0.05).

Pathway maps were created to show genes and compounds responding to UV-B exposure, and heat maps of changes in expression were used to identify enzyme genes and metabolites with notable alterations in flavonoid biosynthesis pathways (Figure 5a). The findings demonstrated that under UV-B stress, naringenin chalcone, dihydrokaempferol, naringenin, epigallocatechin, and epiafzelechin exhibited upregulated tendencies. Additionally, there was a considerable upregulation of the bifunctional dihydroflavonol 4-reductase (DFR) family gene *TRINITY_DN26672_c0_g1_i1-A1* and the chalcone isomerase (CHI) family gene *TRINITY_DN8870_c0_g1_i1-A1*. Consequently, during UV-B stress, pertinent genes in the flavonoid biosynthesis pathway are upregulated, and this controls the upregulation of metabolites. Furthermore, to explore the regulatory mechanism of *RcTRP5* on the flavonoid biosynthesis pathway, correlation analyses were carried out for *RcTRP5* and the above two genes as well as *RcTRP5* and the above metabolites. The results demonstrated that *RcTRP5* exhibited a significantly negative correlation with the key genes and metabolites in the pathway, suggesting that *RcTRP5* regulates the changes in related metabolites by negatively modulating the expression levels of key enzyme genes in the flavonoid biosynthesis pathway.

### 2.6. Building and Examining the Three-Dimensional Configurations of Important Enzymes and Their Non-Covalent Interactions in the R. chrysanthum’s Flavonoid Metabolic Pathway in Reaction to UV-B Stress

The *R. chrysanthum* acetylation proteomics study identified 9371 acetylation sites on 3754 proteins, with quantitative information available for 5063 sites on 1991 proteins. We then conducted a systematic bioinformatics analysis of the proteins containing these quantitatively informative sites.

**Figure 6:**
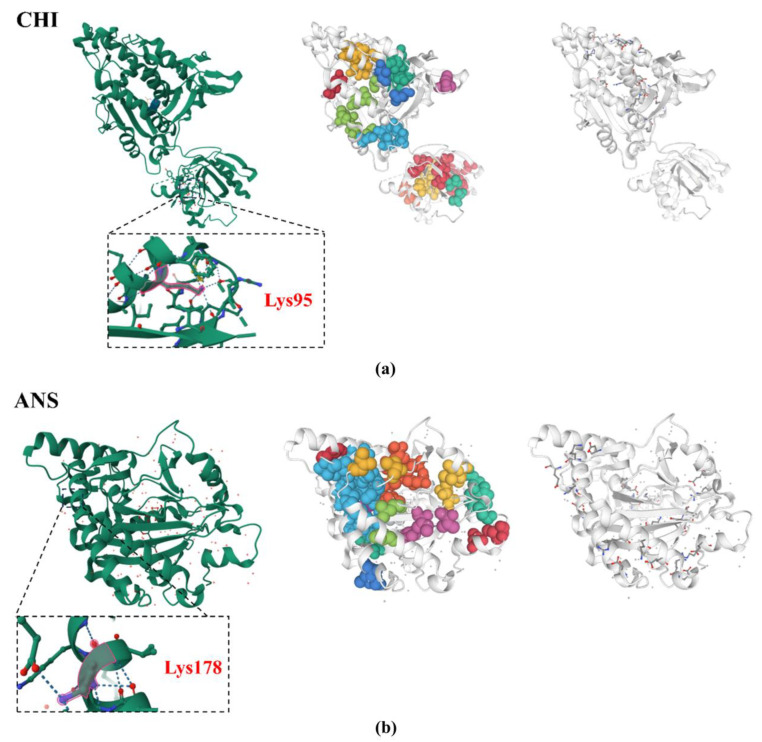
Examination of two important enzymes’ acetylation changes in the *R. chrysanthum* flavonoid biosynthesis pathway: (**a**) from left to right: the three-dimensional architectures of the CHI’s hydrophobic clusters, salt bridges, and acetylation modification sites; (**b**) from left to right: the three-dimensional architectures of the ANS’s hydrophobic clusters, salt bridges, and acetylation modification sites.

To comprehend the characterization and molecular function of the proteins, we examined the acetylation modifications, salt-bridge structures, and water-transporting clusters of the key enzymes in the flavonoid biosynthesis pathway depicted in Figure 5a. The results showed that both CHI and DFR were modified by acetylation under UV-B stress. Specifically, the acetylation modification of CHI was found to be upregulated at the K95 site. Additionally, CHI was shown to have 11 hydrophobic clusters, with the areas of these clusters varying between 24.6 and 1704.6 Å^2^. These clusters contained between two and fourteen residues, with interactions ranging from two to forty-five among the residues. Based on calculations, 74 salt bridges with a kappa (k) value of 0.17 and a fraction of charged residues (FCR) of 0.25 were found in CHI (Figure 6a).

Similarly, there was an upregulation of acetylation modification in ANS, with the modification site at K178. As shown in Figure 6b, ANS had 11 hydrophobic clusters with areas ranging from 35.5 to 3069.6 Å^2^. These clusters contained a total of two to twenty-three residues, with interactions ranging from two to seventy-nine among the residues. Based on calculations, 74 salt bridges with a kappa (k) value of 0.13 and an FCR of 0.30 were found in ANS.

To accurately assess the role of the RcTRP5 transcription factor in the flavonoid biosynthesis pathway, we implemented a parallel acetylation modification study of the RcTRP5 transcription factor. The results revealed that the RcTRP5 transcription factor also undergoes acetylation modification (Figure S1). In addition, we analyzed the interactions between MYB transcription factors and ANS, CHS, DFR, and UVR8. Our findings revealed that all five MYB transcription factors interacted with UVR8, and, similarly, ANS, CHS, and DFR also interacted with UVR8. This suggests that MYB transcription factors are involved in the response of *R. chrysanthum* to UV-B stress (Figure S1).

### 2.7. Examination of the Relationship Between Physiological Indices and RcTRP5 in R. chrysanthum During UV-B Stress

**Figure 7:**
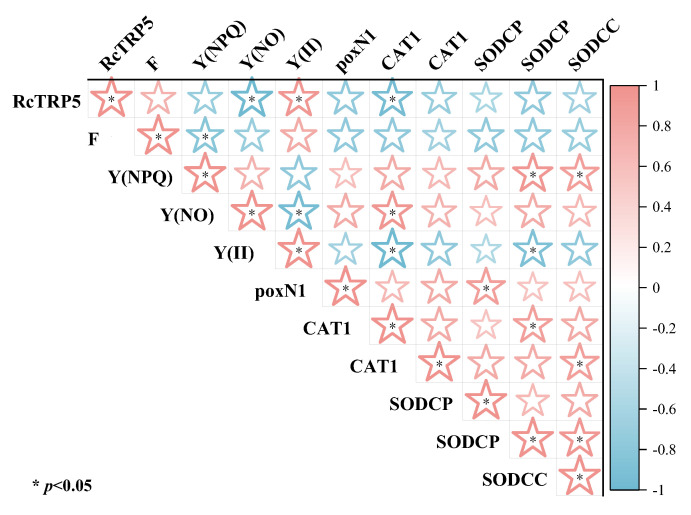
Correlation analysis of *R. chrysanthum’s* antioxidant enzyme systems and photosynthetic parameters under UV-B stress. The stronger the association, the more pinkish the color, and the stronger the correlation, the more bluish the color. For *n* = 3, the data are the mean ± SD. Asterisks denote treatments with significant changes (*p* < 0.05).

Based on the response of *R. chrysanthum* to UV-B stress, the results indicated that *RcTRP5* had significant correlations with Y(NO), Y(II), and CAT. Specifically, *RcTRP5* was negatively correlated with Y(NO) and CAT but positively correlated with Y(II). This suggests that *RcTRP5* may play a role in removing reactive oxygen species from the photosynthetic organs of *R. chrysanthum* by regulating the antioxidant enzyme system. By doing so, it helps maintain the stability of photosynthetic pigments and regulates the transport and distribution of photosynthetic products, thereby enabling the plant to resist UV-B stress (Figure 7).

### 2.8. Creation of a Model Map to Show How the R. chrysanthum Reacts to UV-B Stress

**Figure 8:**
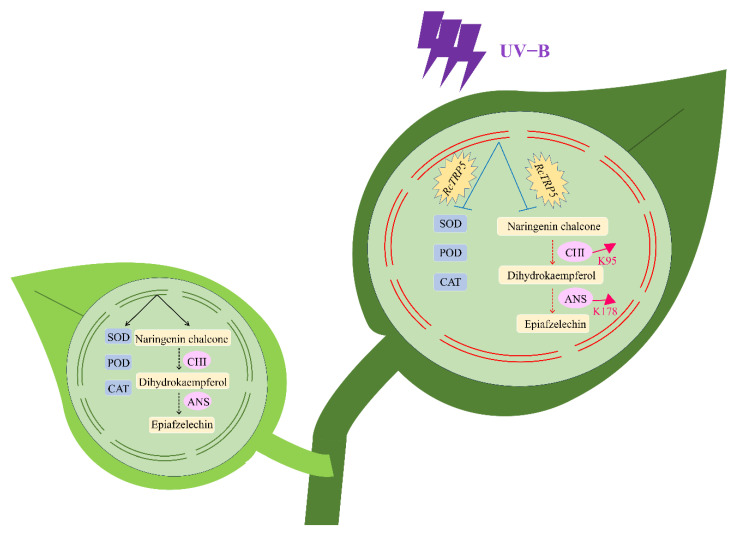
Diagram illustrating the defense mechanisms that *R. chrysanthum* uses against UV-B rays. *R. chrysanthum’s* enzyme systems and flavonoid biosynthesis pathways under normal light and UV-B stress are depicted in the left and right leaves, respectively. The damaging injuries and reactions to UV-B stress in *R. chrysanthum* are shown by the red lines. Acetylation modification sites and their upregulation are indicated by pink arrows. Inhibitory effects are indicated by blue lines.

Under UV-B stress, *R. chrysanthum* exhibited significant decreases in F and Y(II), along with increases in Y(NPQ) and Y(NO). These findings suggest that UV-B stress directly inhibits the plant’s photosynthetic activity, prompting the plant to defend itself by enhancing heat dissipation and antioxidant enzyme activity. *RcTRP5* plays a crucial role in this process by regulating the antioxidant enzyme system, which scavenges excess reactive oxygen species from the plant’s photosynthetic organs. Additionally, the flavonoid metabolism pathway is also vital for *R. chrysanthum’s* resistance to UV-B stress. *RcTRP5* negatively regulates the upregulation of specific genes in the CHI and DFR families, namely, TRINITY_DN8870_c0_g1_i1-A1 and TRINITY_DN26672_c0_g1_i1-A1, which further affects the upregulation of dihydrokaempferol and epiafzelechin in the flavonoid biosynthesis pathway. Specifically, *RcTRP5* negatively regulates the upregulation of the K95 site of CHI and the K178 site of ANS, adversely impacting the upregulation of dihydrokaempferol and epiafzelechin. The increased expression of UV-absorbing compounds such as epiafzelechin and dihydrokaempferol further demonstrates *R. chrysanthum’s* ability to reduce damage from UV-B stress (Figure 8).

## 3. Discussion

As a widely used evaluation method in the research of plant photosynthesis, the chlorophyll fluorescence methodology more accurately captures the photochemical rate of plants in challenging environments [22]. The change in Fv represents the maximum reduction in Q_A_; a decrease in Fv limits the reducing ability of Q_A_ and reduces the amount of light energy that the PSII reaction center can use and convert. This decrease in light energy directly correlates with a decrease in R. chrysanthum photosynthetic activity (Figure 1a). Y(II) represents the ratio of light quanta that are really used to those that are absorbed by the PSII reaction center [23] (Figure 1d). A decrease in the efficiency of electron transfer from water to plastoquinone (Q_A_), or a drop in Y(II), results from the inhibition of the electron transfer pathway caused by damage to the PSII reaction center. *R. chrysanthum* simultaneously develops a non-photochemical quenching mechanism to mitigate the over-excitation and damage to the PSII reaction center; this increases the Y(NPQ), indicating the plants’ photoprotection [24] (Figure 1b). In contrast, Y(NO) represents the plant’s light damage, and as the *R. chrysanthum* under UV-B stress decreased its PSII reaction center activity, Y(NO) naturally increased (Figure 1c).

UVR8 is a UV-B-specific photoreceptor that was first identified from an *Arabidopsis* mutant that is allergic to UV-B. Upon UV-B irradiation, UVR8 dimers undergo monomerization and accumulate in the nucleus, thereby initiating regulatory functions via gene expression and metabolic pathways in plants [25,26]. In our study, we found that UVR8 interacts with MYB transcription factors and key enzymes in the flavonoid metabolic pathway to help cowpea azalea resist UV-B stress. This is similar to the results of a previous study. CmUVR8 regulates the expression of genes involved in flavonoid biosynthesis and UV-B-induced flavonoid accumulation in chrysanthemums [27].

Plants have a reactive oxygen species (ROS) homeostasis system that maintains intracellular homeostasis and can be used to counteract harmful reactive substances (ROS) [28]. Excessive accumulation of ROS in cells can inhibit plant growth and, in severe cases, lead to plant cell death [29]. Plants have developed a comprehensive system of antioxidant enzymes, including POD and SOD, to scavenge excess ROS from the body to prevent overaccumulation [30]. SOD, an enzyme that contains metals, catalyzes the disproportionation process of O^2−^ superoxide to make H_2_O_2_, which can then be used by POD or transformed by CAT into safe H_2_O and O_2_ [31].

Once UV-absorbing substances are produced, photochemical processes occur, which means that the energy of the radiation that reaches each part is decreased by its absorption, hence reducing the direct influence of UV-B radiation on other organelles and functional components of the cell [32]. Research has indicated that flavonoids are not only the primary absorbers of UV-B radiation, but they may also help the antioxidant enzyme system scavenge ROS and effectively limit the harm that UV-B radiation causes to the plant by building up in plants [33]. When UV-B was enhanced, control plants’ foliar surfaces accumulated flavonoids, and their upper leaf surfaces had a greater density of glandular trichomes and stem glands [34]. UV-B radiation significantly influenced plant morphology and secondary metabolite profiles, as shown by higher concentrations of flavonoids, hydroxycinnamic acid, and carotenoid compounds as well as increased dry weight and leaf area [35]; this completely aligns with the test’s outcomes. The results of this experiment showed a considerable increase in the expression of quercetin-3-O-arabinoside, gallocatechin, 6-methoxyflavone, kaempferol-3-O-arabinoside, butin (Figure 2), and naringenin chalcone under UV-B stress treatment (Figure 5a). Based on the aforementioned findings, it is evident that plants enhance the number of UV-absorbing compounds in their bodies to protect themselves from UV-B radiation. This may be an adaptive response of the plants to the increased UV-B radiation.

*RcTRP5* belongs to the *R. chrysanthum* MYB gene. MYB transcription factors are implicated in many abiotic stress responses, including drought, extremes of temperature, and UV exposure [36]. Additionally, previous research has shown that rice’s MYB family genes have a role in the drought stress response; for example, overexpressing *OsMYB2* in rice plants can increase their resistance to drought [37]. The plant-specific transcriptional regulator known as telomere repeat-binding protein (TRB) binds two DNA-binding structural domains, the GH1 structural domain, which binds to the junction DNA and is shared with the H1 histone, and the Myb/SANT structural domain, which specifically recognizes the telobox DNA-binding site motif. TRB4 and TRB5 share a common TRB motif but differ from the TRB1–3 motif and appear to have an earlier phylogenetic origin than TRB1–3. Studies have shown that TRB4 and TRB5 regulate plant development and gene expression [38]. This suggests that OsTRFL1 is an important factor in the maintenance of telomeres and normal rice development [39]. This work revealed that *RcTRP5* functions as a negative regulator, first suppressing the expression of UV-B stress-responsive genes in *R. chrysanthum* and subsequently suppressing the expression of important flavonoids.

CHI is the first recognized flavonoid synthesis-related enzyme, and in the flavonoid synthesis pathway, chalcone isomerase is responsible for the conversion of chalcone to dihydroflavonoids, an important step in flavonoid biosynthesis. It was found that chalcone isomerase-like protein (CHIL) binding to CHS enhances 2′,4,4′,6′-tetrahydroxychalcone (THC) production and reduces *p*-coumaroyltriacetic acid lactone (CTAL) formation, thereby correcting mixed CHS catalysis and contributing to an efficient substrate influx from the phenylpropane pathway to the flavonoid pathway [40]. ANS is one of the key enzymes involved in the anthocyanin biosynthesis pathway, contributing to the maintenance of the stability and function of the photosynthetic system by regulating anthocyanin content and distribution. An overexpression of ANS in rice mutants results in novel transgenic rice with a flavonoid mixture and enhances antioxidant potential [41].

Different environmental conditions are often triggers for regulatory mechanisms. Post-translational modifications are an important aspect of plant response to stress [42]. It was found that HY5 directly binds to the HsfA2 promoter’s G box motif in Arabidopsis, where it works with histone deacetylase 9 (HDA9) to suppress the expression of the gene [43]. Furthermore, we found that the flavonoid metabolism pathway acetylated both DFR and ANS under UV-B stress and that the acetylation sites of both proteins were markedly upregulated. The above studies suggest that flavonoid accumulation leads to histone acetylation.

When facing abiotic stress, plants will trigger a series of complex physiological and molecular responses to adapt to their environment. TRP5, as a transcriptional regulator, may form a complex network with other transcription factors to co-regulate gene expression [38,44]. Through high-throughput sequencing and bioinformatics analysis, the interactions between TRP5 and other transcription factors and their regulatory networks can be revealed. With the continuous development of multi-strategy genomics technology, the function and regulatory mechanism of TRP5 can be investigated in a wider scope, and the specific role of TRP5 in gene expression regulation can be studied in depth, including its recognition and binding to specific gene promoters. For example, the expression pattern and functional changes of TRP5 in individual cells can be studied using single-cell sequencing technology; the spatial distribution and functional differences of TRP5 in specific tissues or organs can be studied using spatial transcriptomics technology.

## 4. Materials and Methods

### 4.1. Plant Material, Growing Conditions, and Treatments

In this experiment, *R. chrysanthum* originating from Changbai Mountain (40.10° N, 100.10° E) was cultivated on 1/4 MS solid medium in an artificial intelligence incubator maintained at 25 °C during the day and 18 °C at night, with a 10 h light/14 h dark cycle and a photon flux density of 50 µmol·m^−2^·s^−1^. Six seedlings of uniform growth were selected and divided into two groups, each with three biological replicates, for experimental treatment.

PAR + UV-B stress treatment group: From the six plants, three morphologically identical *R. chrysanthum* with a uniform genetic background were randomly selected to form the UV-B stress treatment group (UV-B). Their culture flasks were equipped with a 295 nm filter (Edmund Optics Filter Long 2 in SQ, Barrington, NJ, USA) and subjected to UV-B radiation for 48 h while simultaneously being exposed to PAR + UV-B light.

Control treatment group: The remaining three morphologically identical *R. chrysanthum* with a uniform genetic background served as the control group (Control). These were placed under a 400 nm filter (Edmund Optics Filter Long 2 in SQ, Barrington, NJ, USA) and exposed solely to PAR light for 48 h.

Warm-white fluorescent bulbs (Philips, T5 14W, Amsterdam, The Netherlands) served as the source of photosynthetically active radiation (PAR). UV-B fluorescent tubes (Philips, Ultraviolet-B TL 20W/01 RS, Amsterdam, The Netherlands) were used as the light source for UV-B radiation. Monitoring was conducted using a UV intensity meter (model ST-513, SHH, New Taipei City, China) and an illuminance meter (model Tes-1339 Light Meter Pro, TES Electrical Electronic Corp., Taipei, China). The effective illuminance for the UV-B stress-treated group was 2.3 W/m^2^ for UV-B, whereas the control group was exposed to 50 µmol·m^−2^·s^−1^ of PAR.

### 4.2. Determination of Chlorophyll Fluorescence Parameters

Imagine-PAM Maxi (Walz, Effeltrich Version, Rohrdorf, Nuremberg, Germany) was used to calculate the parameters for chlorophyll fluorescence, and leaves were dark-acclimated for 30 min before testing to guarantee that the major electron acceptor Q_A_ was in its maximum oxidized condition. The initial fluorescence Fo was measured under light, and the saturation pulse light was used for excitation to measure the maximum fluorescence Fm. Under actinic light, the *R. chrysanthum* was excited for photosynthesis to obtain the fluorescence value of the Kautsky-induced maximum fluorescence Fp. The parameters calculated from the final measurements were variable fluorescence F, quantum yield Y(NPQ) for modulatable quenching, quantum yield for non-photochemical quenching: [Y(NO) = 1/(NPQ + 1 + qL(Fm/Fo-1))], and actual photo-synthetic efficiency Y(II).

### 4.3. Identification of R. chrysanthum Metabolites Using UPLC–MS/MS-Based Method

Metware Biotechnology Co. (Wuhan, China) extensively conducted targeted metabolomics tests of bovine leatherjacket in this investigation. The following protocols were followed for metabolomic analyses:

*R. chrysanthum* samples were vacuum-freeze-dried using a Scientz-100F lyophilizer and subsequently crushed into powder at 30 Hz for 1.5 min. Precisely 50 mg of the powdered sample was mixed with 1200 μL of a pre-cooled (−20 °C) 70% methanol aqueous solution containing an internal standard. The mixture was centrifuged at 12,000× *g* rpm for 3 min to obtain the supernatant, which was then filtered through a 0.22 μm microporous membrane and transferred to an injection vial for UPLC–MS/MS analysis. The analysis was conducted using a tandem mass spectrometry system (https://sciex.com.cn/ (accessed on 9 September 2024)) and a UPLC–ESI–MS/MS system (UPLC, ExionLC™ AD, https://sciex.com.cn/ (accessed on 11 September 2024)). The entire experimental procedure adhered strictly to the standardized protocol outlined previously [21].

Triple quadrupole mass spectrometry in MRM mode was employed for chemical quantification. Compound categorization was based on secondary spectrum information using the Metware database. Differential metabolites were identified with VIP > 1 using orthogonal partial least squares discriminant analysis (OPLS-DA), with FC > 1.2 indicating significant upregulation and FC < 0.67 indicating significant downregulation. Metabolite enrichment and classification were conducted based on the KEGG database.

### 4.4. Analysis of the Transcriptome of R. chrysanthum

BGI Genomics Co., Ltd. (Shenzhen, China), conducted the transcriptomics assay of the *R. chrysanthum* in this investigation. The precise experimental analysis protocol was as follows:

Total RNA from *R. chrysanthum* was first enriched for mRNA. Following disruption with interruption buffer and reverse transcription using N6 primers, a double-stranded DNA molecule was synthesized. The 5′ end of this molecule was then flattened and phosphorylated, while the 3′ end was adenylated (A-tailed) to create a sticky end. A raised “T” junction was attached to the 3′ end. Subsequently, the ligation product was amplified using specific primers via polymerase chain reaction (PCR). Finally, sequencing was performed online.

Clean reads are produced during the sequencing process when polluted joints, reads with a high number of unknown bases N, and reads with poor quality are removed. Using the assembled clean reads, the Unigene was subjected to functional annotation and SSR detection. Subsequently, the expression levels of the UV-B stress-treated group and the control group were computed using the All-Unigene, and the genes that showed differential expression were analyzed using clustering and functional enrichment techniques.

Clean reads were aligned to the genomic sequence using Bowtie2, and gene expression levels were calculated using RSEM to identify DEGs in *R. chrysanthum* in response to UV-B stress. DEGs were defined as those with a Q-value (adjusted *p*-value) < 0.05.

### 4.5. Proteomic Analysis of R. chrysanthum

#### 4.5.1. Extraction of Proteins

After grinding the sample into a powder using liquid nitrogen, it was transferred to a 5 mL centrifuge tube. The powder was lysed sonically after adding 4 volumes of phenol extraction buffer containing 10 mM dithiothreitol, 1% protease inhibitor, and 1% phosphatase inhibitor. Phenol equilibration was achieved by adding an equal volume of Tris, followed by centrifugation at 4 °C and 5500× *g* for 10 min. The supernatant was discarded, and the protein precipitates were allowed to precipitate overnight with 5 volumes of 0.1 M ammonium acetate/methanol. Next, the precipitates were washed with methanol and then acetone. The final precipitate was re-solubilized with 8 M urea, and the protein concentration was determined using a BCA kit (Beyotime Biotechnology (Shanghai, China)).

#### 4.5.2. Trypsin Digestion

To reduce the protein solution to a final concentration of 5 mM, dithiothreitol was added, and it was reduced for 30 min at 56 °C. Subsequently, iodoacetamide was added to a final concentration of 11 mM and allowed to sit at room temperature in the dark for 15 min. The sample’s urea concentration was finally diluted to be less than 2 M. A mass ratio of 1:50 (trypsin/protein) should be added, and the protein should hydrolyze overnight at 37 °C. After adding trypsin, the protein to trypsin mass ratio was 1:100, and the digestion process was allowed to proceed for four hours.

#### 4.5.3. TMT Labeling

Using Strata X C18 (Phenomenex, Torrance, CA, USA), trypsinized peptides were desalted before being vacuum-freeze-dried. The TMT kit (Thermo Fisher Scientific (Shanghai, China)) instructions were followed to solubilize the peptides in 0.5 M TEAB, label them, mix them, desalt them, and vacuum-freeze-dry them.

#### 4.5.4. HPLC Fractionation

Using an Agilent 300Extend C18 column (5 μm particle size, 4.6 mm inner diameter, and 250 mm length), peptides were graded using high-pH reverse HPLC.

#### 4.5.5. LC–MS/MS Analysis

Using an EASY-nLC 1000 ultra-high-performance liquid chromatography (UHPLC) (Thermo Fisher Scientific (Shanghai, China)). system, peptides were dissolved in liquid chromatography mobile phase A (0.1% (*v*/*v*) formic acid aqueous solution) and then separated. After being separated using a UPLC machine, the peptides were fed into an NSI ion source to cause ionization, and Q Exactive mass spectrometry was used to examine the results. An Orbitrap with high resolution was used to identify and evaluate the parent ions of the peptides as well as their secondary fragments.

#### 4.5.6. Database Search

MaxQuant (v1.5.2.8) was used to retrieve secondary mass spectrometry data. To account for the false positive rate (FDR) resulting from chance matches, inverse libraries were incorporated into the search configuration. Additionally, common contaminant libraries were added to the database to eliminate the impact of contaminating proteins on the ID outcomes. The final classification of significantly upregulated proteins was *p* < 0.05 and FC > 1.5.

### 4.6. Acetylation Modification Proteomics Assay of R. chrysanthum

#### 4.6.1. Extraction of Proteins

Samples were ground to a powder with liquid nitrogen and transferred to a 5 mL centrifuge tube. We added 4 mL of phenol extraction buffer containing 10 mM dithiothreitol, 1% protease and phosphatase inhibitors, and sonic lysis. We added an equal volume of *Tris* and centrifuged at 4 °C and 5500× *g* for 10 min until phenol equilibrium was achieved. The supernatant was discarded, and 5 volumes of 0.1 M ammonium acetate/methanol precipitated the protein overnight. After washing with methanol and acetone, 8 M urea was re-solubilized, and the protein concentration was measured using a BCA kit (Beyotime Biotech, Shanghai, China) [21].

#### 4.6.2. Trypsin Digestion

To lower the protein solution concentration to 5 mM, we added dithiothreitol and left it at 56 °C for 30 min. We added iodoacetamide to 11 mM and left it at room temperature in the dark for 15 min. We diluted the concentration of urea to less than 2 M, added trypsin (trypsin/protein = 1:50, mass ratio), and hydrolyzed it at 37 °C overnight. The ratio was later adjusted to 1:100, and digestion was continued for four hours.

#### 4.6.3. HPLC Fractionation

NanoElute ultra-high performance liquid chromatography (UHPLC) technology was used to separate the peptides after they were solubilized in liquid chromatography mobile phase A. After being separated using a UHPLC system, the peptides were fed into a capillary ion source to be ionized, and tims-TOF Pro mass spectrometry was used to examine the results. High-resolution TOF was utilized to detect and analyze the parent ions of the peptide and their secondary fragments, with the ion source voltage set at 2.0 kV. Parallel accumulation–serial fragmentation (PASEF) mode was utilized for data gathering, and the secondary MS scan range was set to 100–1700. With parent ion charges in the range of 0–5, one primary mass spectrum and ten secondary spectra were obtained in PASEF mode. To prevent repeated parent ion scans, the tandem mass spectral scan’s dynamic exclusion period was adjusted to 30 s.

#### 4.6.4. Database Search

MaxQuant 1.6.6.0 was used to retrieve data from secondary mass spectrometry. To account for the false positive rate (FDR) resulting from chance matches, inverse libraries were incorporated into the search parameter settings. Additionally, common contaminant libraries were added to the database to eliminate the impact of contaminating proteins on the identification outcomes. Finally, it was decided that acetylation changes had considerably increased the expression of proteins with *p* < 0.05 and FC > 1.5.

### 4.7. Data Analysis

In each of the aforementioned studies, three biological replicates were used in a fully randomized trial design. Data on rhododendron bovine fluorescence parameters and omics parameters were represented as the mean ± standard deviation, and univariate differences across samples were analyzed for significance (*p* < 0.05) using IBM SPSS Statistics 26 (IBM Corporation, New York, NY, USA).

## 5. Conclusions

In this study, we conducted a comprehensive analysis of physiological indices, transcriptomics, metabolomics, proteomics, and acetylation proteomics to investigate the response of *RcTRP5* to UV-B stress. We found that *RcTRP5* expression was downregulated, while the acetylation of the RcTRP5 transcription factor at K68 was upregulated, leading to an increased expression of SOD, POD, and CAT. The visualization of the flavonoid biosynthesis pathway revealed that *RcTRP5* indirectly negatively regulates the acetylation of CHI and ANS, thereby accelerating flavonoid biosynthesis. Our findings establish a theoretical framework for enhancing UV resistance and improving plant quality and provide insights into the MYB transcription factor control of flavonoid production in *R. chrysanthum* under UV-B stress.

## Data Availability

The datasets generated during and/or analyzed during the current study are available from the corresponding author on reasonable request.

## Supplementary Materials

The following supporting information can be downloaded at https://www.mdpi.com/article/10.3390/ijms252413383/s1.
