# Supplementary material for: The Molecular Mechanism Regulating Flavonoid Production in Rhododendron chrysanthum Pall. Against UV-B Damage Is Mediated by RcTRP5
Source: Int J Mol Sci. 2024 Dec 13;25(24):13383. doi: 10.3390/ijms252413383 (PMC11677096; doi:10.3390/ijms252413383)
Supplement: Supplementary file 1 [file ijms-25-13383-s001.zip › Figure. S1.pdf]

E1.11.1.7

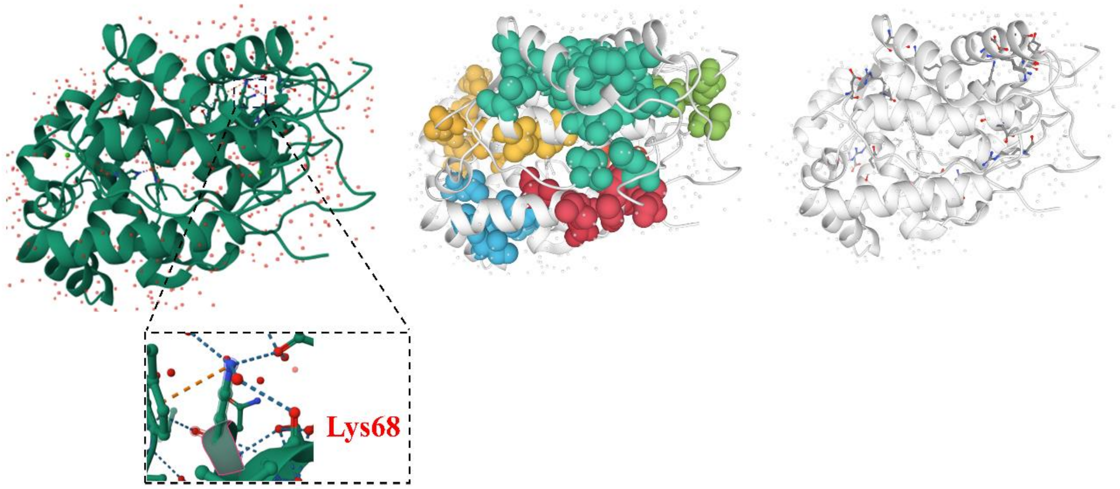

Figure. S1 From left to right, the three-dimensional architectures of the E1.11.1.7's hydrophobic clusters, salt bridges, and acetylation modification sites are shown.

A correlation analysis was conducted using the transcription factor *RcTRP5* responding to UV-B stress in *R. chrysanthum*, 13 UVR8s, and the enzymes and metabolites with significant changes in the flavonoid metabolic pathway. There was a significant negative correlation between *RcTRP5* and UVR8-1, UVR8-5, a significant positive correlation between UVR8-1 and CHS1, CHI3, a significant positive correlation between UVR8-5 and Naringenin chalcone, and a significant positive correlation between UVR8-7 and ANS17, CHS. In addition, UVR8-8 showed a positive correlation with Naringenin. Overall, these results suggest that UVB8-1, UVB8-5 may be regulated by *RcTRP5* and involved in flavonoid biosynthesis in response to UV-B stress.

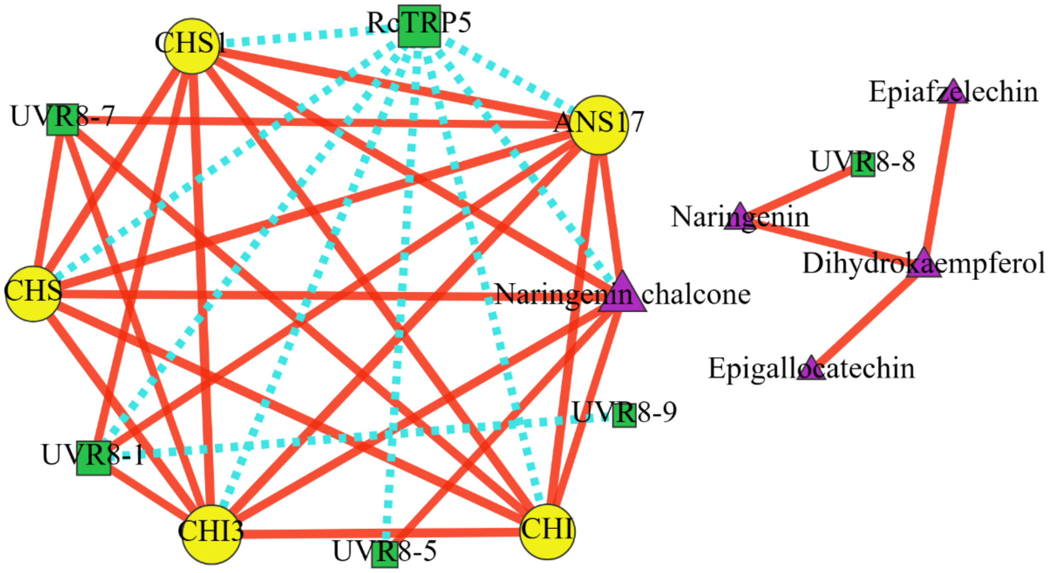

Figure. S2 Correlation analysis between UVR8 and *RcTRP5*, key enzymes and key metabolites in the flavonoid biosynthetic pathway. Where, red solid line indicates positive correlation and blue dashed line indicates negative correlation.
